# An Adult With Undifferentiated Embryonal Sarcoma of the Liver: A Case Report of a Rare Encounter

**DOI:** 10.7759/cureus.45018

**Published:** 2023-09-11

**Authors:** Rajmohan Rammohan, Melvin Joy, Sai Greeshma Magam, Achal Patel, Sai Reshma Magam, Dilman Natt, Jiten Desai, Susan Bunting, Paul Mustacchia

**Affiliations:** 1 Gastroenterology, Nassau University Medical Center, East Meadow, USA; 2 Internal Medicine, Nassau University Medical Center, East Meadow, USA; 3 Gastroenterology and Hepatology, Nassau University Medical Center, East Meadow, USA

**Keywords:** hamartomas, mesenchymal, sarcomatoid, carcinosarcomas, liver's mesenchymal tissue, undifferentiated embryonal sarcoma

## Abstract

Undifferentiated embryonal sarcoma of the liver (UESL) is a rare, aggressive tumor mainly found in children but can also appear in adults. Its diagnosis in adults remains a conundrum; it is often identified late due to its non-specific symptoms and resemblance to benign lesions. A comprehensive treatment regimen involving surgical intervention, chemotherapy, and possibly radiation significantly boosts survival rates. Imaging often yields inconclusive outcomes, further complicating the diagnostic process.

Here, we report the case of a 28-year-old female diagnosed with UESL, emphasizing the need for timely intervention. Undifferentiated embryonal sarcoma of the liver requires differentiation from a variety of hepatic tumors in adults. Though there are no distinctive characteristics to differentiate UESL from other hepatic masses, its morphology and immunohistochemical profiles significantly vary. The staging often reveals UESL as a large, well-defined mass with the potential for diverse differentiation. Its prognosis has been considerably improved with the advent of multidisciplinary treatment. Surgical resection remains a cornerstone, often combined with chemotherapy. While pediatric cases exhibit better overall survival rates than adults, outcomes heavily depend on the chosen treatment regimen. A combination of chemotherapy and complete tumor removal has been found to significantly elevate survival chances.

Disease recurrence remains a challenge and is influenced by treatment strategy. In conclusion, the diagnosis and treatment of UESL are fraught with challenges, particularly in adults. A multidimensional approach, combining various therapies, is paramount for better outcomes. Continuous research and enhanced awareness are crucial for improving diagnostic precision and treatment outcomes for UESL patients.

## Introduction

Undifferentiated embryonal sarcoma of the liver (UESL) is a rare and aggressive tumor originating from the liver's mesenchymal tissue [1]. It is predominantly found in children aged six to 10, but it can also occur in adults [1]. An UESL is highly metastatic and notorious for its non-specific symptoms, such as an abdominal mass, making it a diagnosis of exclusion [1,2]. This means that UESL is often identified only after other possibilities are ruled out, making the process complex and potentially delayed. In adults, UESL accounts for less than 1% of primary liver tumors, and its diagnosis is challenging due to the lack of specific clinical features for differentiation [3]. Treatment is possible if diagnosed promptly, and a comprehensive approach involving surgery, chemotherapy, and radiation can significantly enhance long-term survival rates. However, imaging studies like ultrasound, CT, or MRI often provide inconclusive results, as UESL typically manifests as a large cystic liver mass [3]. The tumor's resemblance to benign lesions frequently leads to misdiagnoses, resulting in a delay that may cause the condition to present past childhood. Because of these nonspecific characteristics, UESL should always be considered in the differential diagnosis, even in adults with large liver tumors [3]. An illustrative example of this disease's complexity and uniqueness is this case report of a 28-year-old woman from El Salvador diagnosed with UESL. Her situation highlights the need for awareness and early intervention to manage this rare, treatable, and potentially deadly liver tumor.

## Case presentation

Our patient, a 28-year-old Hispanic female from El Salvador, experienced two days of severe epigastric pain, rated at 10/10, radiating to different quadrants of her abdomen, accompanied by nausea, anorexia, and chills. Medical history revealed family instances of liver and stomach cancer. Upon examination, her upper abdomen was extremely tender, and laboratory tests indicated leukocytosis with eosinophilia and normal hepatic and renal function. A CT and an MRI scan revealed a complex cystic mass in the left hepatic lobe, prompting recommendations to rule out various conditions such as liver abscess, hydatid cyst, and amebic abscess. Treatment began with proton-pump inhibitor (PPI) therapy, pain medications, and antibiotics. She showed improvement, leading to a consultation for an elective robot-assisted left hepatectomy. Following discharge and outpatient follow-up, a repeat CT scan displayed increased cystic mass size and visible tumor neovascularity. Readmitted for surgical intervention, a partial liver resection was conducted, with pathology identifying the mass as an embryonal sarcoma of the liver, measuring 10 x 7.5 x 3 cm with negative margins. The patient was started on chemotherapy with doxorubicin/ifosfamide and vincristine/dactinomycin/cytoxan.

**Figure 1:**
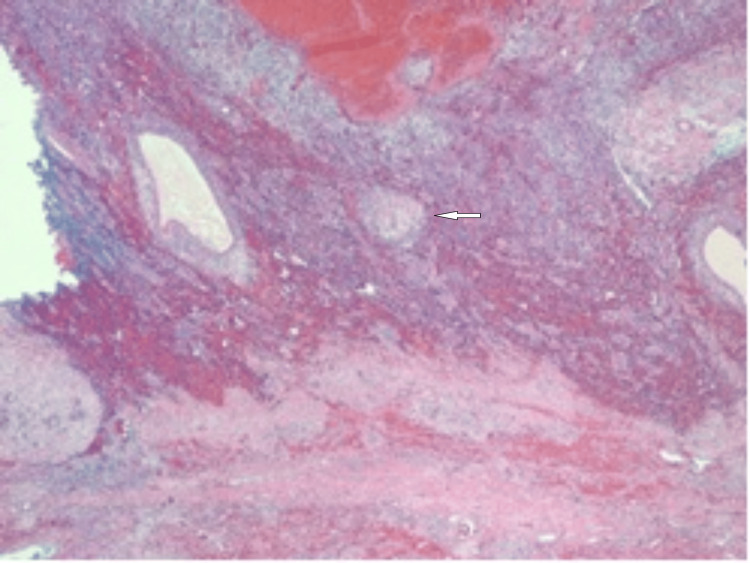
A hemorrhagic and cystic lesion of the liver (white arrow; 2x field)

**Figure 2:**
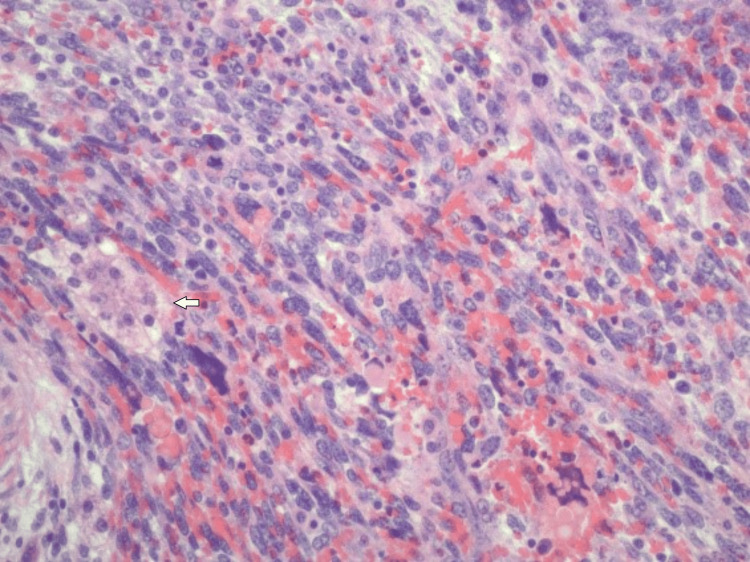
Spindle neoplastic cells and pleomorphic giant tumor cells (white arrow, 10x field)

**Figure 3:**
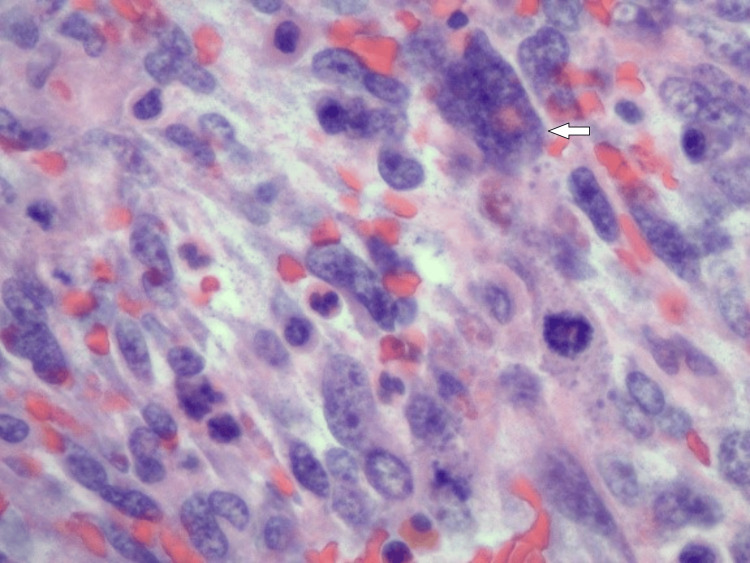
Pleomorphic giant tumor cells with intracellular eosinophilic globules (white arrow). Frequent cellular mitosis is also noted.

Immunohistochemical stains supported the diagnosis, with negative results for several markers and nonspecific results for the FLI1 stain (Figures 1-3). The immunohistochemistry (IHC) stain was also negative for MDM2. This case demonstrates a complex medical journey from the initial presentation to the final diagnosis of a rare liver condition. The coordination of various medical specialists and the utilization of numerous diagnostic tools were vital in ultimately identifying and treating this serious liver ailment.

## Discussion

Differential diagnosis 

In the context of adults, distinguishing UESL involves discriminating it from carcinosarcomas, sarcomatoid or spindle-cell carcinomas, mesenchymal hamartomas, mixed hepatoblastomas displaying spindle-cell characteristics, angiomyolipoma, and a variety of other sarcomas, encompassing malignant fibrous histiocytoma, leiomyosarcoma, osteosarcoma, angiosarcoma, liposarcoma, melanoma, rhabdomyosarcoma, or malignant schwannoma [4,5]. While no distinct attributes besides size differentiate UESL from other hepatic masses in adults, the morphology and immunohistochemical profiles of different hepatic masses significantly diverge from those of UESL. Furthermore, the immunohistochemical profile of UESL lacks specificity and diagnostic value, showcasing evidence of widely varying differentiation [6].

Staging 

Predominantly situated in the right lobe, UESL can also manifest simultaneously in the left lobe or bilaterally [6]. Macroscopically, UESL typically presents as a sizable, solitary, well-defined mass with variable areas of hemorrhage, necrosis, and cystic degeneration. Under the microscope, the tumor comprises loosely arranged, medium-large spindles, pleomorphic oval and stellate cells with poorly defined boundaries, and giant cells displaying severe atypia. Despite its uncertain pathological origin, ultrastructural and immunohistochemical analyses have revealed diverse differentiations such as fibroblastic, histiocytic, lipoblastic, myofibroblastic, rhabdomyoblastic, and leiomyoblastic differentiation [5]. Vimentin is widely positive in most UESL cases, along with focal positivity for a1-AT, cytokeratin, desmin, α-smooth muscle actin (SMA), muscle-specific actin, CD68, myoglobin, non-specific enolase, S100, and CD34. This suggests that embryonal sarcomas fall into the 'undifferentiated' sarcoma category due to their potential for partial differentiation [4,6]. Additionally, genetic mutations are complex and heterogeneous. Mutations in p53 and the absence of the telomerase catalytic subunit, human telomerase reverse transcriptase, could contribute to its unfavorable biological behavior [7].

Treatment

Primarily affecting pediatric patients, knowledge about UESL prognosis largely derives from pediatric cases. Historically, the outlook for this tumor was grim until a multidisciplinary treatment approach was established. The five-year overall survival rate for children is approximately 86% [1,8].

Surgical resection remains pivotal in treating these patients. Like most tumors, negative margins are sought in surgical procedures. However, some studies have suggested that positive margins might not significantly impact outcomes due to chemotherapy's potential to eliminate residual tumors. Given the tumor's rarity, ongoing research will likely clarify the role of margin status [1,8].

Chemotherapy is now commonly combined with surgery to treat UESL, significantly improving patient outcomes. However, the specific chemotherapy regimen varies among institutions and oncologists due to the need for a standardized approach [1,8-10]. In select cases, radiation therapy has been used either in metastatic scenarios or as a preventive measure against tumor recurrence. Yet the efficacy of radiotherapy remains uncertain, requiring further investigation before a consensus can be reached. Unresectable tumors have prompted the consideration of orthotopic liver transplants, which may offer benefits in certain situations [1,8-13]. Nevertheless, the data on this treatment approach are limited.

Disease recurrence and prognosis

Patients diagnosed with UESL typically have a challenging prognosis [14]. On average, their survival duration is about 29 months [15]. Within the first year, the survival rate stands at 61%, dropping slightly to 55% in the second year [16]. Survival diminishes significantly, to just 12 months, for those who have only partial tumor removal and do not undergo chemotherapy [15]. However, in instances where chemotherapy was evaluated, there was a 62% rate of tumor reduction, even in instances of significant necrosis [17]. This data suggests that a combination of chemotherapy and tumor removal boosts survival chances [15]. On the other hand, only partial tumor removal leads to less favorable outcomes [15].

Undifferentiated embryonal sarcoma of the liver is more prevalent among children. Interestingly, the pediatric population shows better overall survival rates compared to adults: 86.8% for the first year, 75.4% for three years, and 71.1% for five years [18]. Metastatic UESL cases demonstrate a five-year survival rate of 53.1%, while non-metastatic cases fare better at 76% [18]. Surgical tumor removal has also shown promising results, with a five-year survival rate of 79.1% compared to a mere 36.3% for non-surgical treatments [18]. Similarly, chemotherapy recipients have a five-year survival rate of 74.9%, noticeably higher than the 50.3% for those who did not undergo chemotherapy [18].

A study involving 25 UESL patients found a 32% recurrence rate [15]. When broken down further, among the 13 patients who had complete tumor removal and chemotherapy, 23% faced recurrence [15]. In contrast, 42% of the 12 patients who had only the tumor removed experienced a return of the disease [15]. This information underscores the fact that the course and recurrence of UESL are heavily influenced by the chosen treatment method [15].

For those who had their tumors entirely removed but did not have chemotherapy, a sizable 42% experienced a forceful recurrence, typically within eight months post-treatment [15]. However, with the addition of subsequent chemotherapy, recurrence dropped to 23%, with an average recurrence period of 28 months post-treatment [15]. Finally, the role of interventional radiology in UESL treatment is growing, aiming to lessen the side effects of systemic chemotherapy and shrink tumors before surgery [16].

## Conclusions

Undifferentiated embryonal sarcoma of the liver remains a challenging and complex diagnosis, given its rarity and nonspecific presentations. This difficulty is further amplified in adults, where the disease is even less common. The importance of timely diagnosis and comprehensive treatment, spanning surgical resection, chemotherapy, and, in some cases, radiation, is evident in the stark differences in survival rates and outcomes. Pediatric cases show more promising survival figures than adults, underscoring the disparities in prognosis. Furthermore, the role of various therapies, from conventional treatments to emerging interventional radiology, suggests a multidimensional approach is crucial. Continuous research and heightened awareness are essential to enhancing diagnostic accuracy and outcomes for UESL patients.
